# The future of extreme climate in Iran

**DOI:** 10.1038/s41598-018-38071-8

**Published:** 2019-02-06

**Authors:** Saeid Ashraf Vaghefi, Malihe Keykhai, Farshid Jahanbakhshi, Jaleh Sheikholeslami, Azadeh Ahmadi, Hong Yang, Karim C. Abbaspour

**Affiliations:** 1 Eawag, Swiss Federal Institute of Aquatic Science and Technology, Duebendorf, Switzerland; 2 Department of Civil Engineering, Isfahan University of Technology, Isfahan, Iran; 3 Department of Watershed Management Engineering, Yazd University, Yazd, Iran; 4 Department of Environmental Science, University of Basel, Basel, Switzerland

**Keywords:** Climate sciences, Hydrology

## Abstract

Iran is experiencing unprecedented climate-related problems such as drying of lakes and rivers, dust storms, record-breaking temperatures, droughts, and floods. Here, we use the ensemble of five high-resolution climate models to project maximum and minimum temperatures and rainfall distribution, calculate occurrences of extreme temperatures (temperatures above and below the historical 95th and 5th percentiles, respectively), analyze compound of precipitation and temperature extremes, and determine flooding frequencies across the country. We found that compared to the period of 1980–2004, in the period of 2025–2049, Iran is likely to experience more extended periods of extreme maximum temperatures in the southern part of the country, more extended periods of dry (for ≥120 days: precipitation <2 mm, Tmax ≥30 °C) as well as wet (for ≤3 days: total precipitation ≥110 mm) conditions, and higher frequency of floods. Overall, the combination of these results projects a climate of extended dry periods interrupted by intermittent heavy rainfalls, which is a recipe for increasing the chances of floods. Without thoughtful adaptability measures, some parts of the country may face limited habitability in the future.

## Introduction

Floods and droughts have been occurring all the times in the past, but previous research shows that these occurrences are happening in increasing rates and that the changes can be dominantly due to anthropogenic activities^1^. A sequence of processes due to increasing greenhouse gasses, could be summarized as (*i*) increases in air temperature and its capacity to hold more water^2,3^, (*ii*) accelerated and irreversible melting of permafrost^4^, glaciers^5^, and ice caps^6^ adding more water into the atmosphere, and (*iii*) increases in plant biomass and evapotranspiration^7,8^. The net result is a transfer of more surface water into an atmosphere capable of holding more water. More substantial accumulation of atmospheric water will cause a higher frequency of high-intensity and short-duration precipitations^3,9–11^, significantly increasing the chances of flooding in different parts of the world.

In recent decades, observed climate data clearly show a warming trend in many parts of the world, resulting in a wide range of climatic impacts^3,9,11–13^. In Iran, a country dominated by an arid and semi-arid climate, significant climate anomalies have been observed^14^. In combination with management related issues, Iran has been confronted with many disasters from shrinking a significant number of lakes and river, to land subsidence, floods, and droughts. Lake Urmia - the largest lake in the Middle East and one of the world’s largest hypersaline lakes - has significantly shrunk^15^ and given the status quo, it may completely dry up in 6–9 years^16^. Hamun lake in the east of Iran, Parishan and Shadegan lakes in the south^17,18^, and Zayandeh-Rud river in the center of Iran^19,20^ are also at risk of disappearing due to mismanagement and climate change. Iran also has extremely critical conditions in groundwater resources because of overexploitation, and the country ranks among the top groundwater miners in the world^21^. In this critical water condition, increasing frequency of floods causes severe damages to different levels of water infrastructure, economy, and society at large. From 2015 to 2018, approximately six major floods occurred in unexpected regions located in arid and semiarid parts of the country^22^. In addition, floods in the northern edge of the country often cause substantial damage. The worst flooding disaster occurred on August 2001, where a once in 200-year-flood, affected more than 27,000 people, rendered 10,000 homeless, and killed about 247 people in Golestan province in northern Iran^23,24^.

In the studies of the impact of climate change, most previous projections have been conducted at the decadal time scale to show the trend of changes in temperature and precipitation at the mid to end of the 21^st^ century^25–29^. However, extremely dry and wet periods, as well as floods in intra-annual temporal scale in arid and semi-arid countries, like Iran, have not been thoroughly studied, even though their impacts have been severe. Recent studies of extreme events have mostly focused on single-driver climate indices, such as annual precipitation, maximum one-day precipitation, number of days above a certain threshold, annual frequency of warm days and nights, etc., in arid and semi-arid regions^30–33^. However, there are disagreements on the value and frequency of these indices across the country attesting to the significant uncertainties in the past and future climate data and the period of study.

Although the risk of extreme precipitation or temperature events may extend over a large geographic area, the vulnerability to flooding or drought events is a highly local phenomenon. Hence, while commonly used indices mentioned above are useful in predicting the extreme trends, they are of little use for assessing local floods and droughts. In this article, we look at compound extremes (i.e., the simultaneous occurrence of multiple extremes) of dry and wet periods in Iran and also identify past floods and the associated climate conditions (in terms of duration, intensity, and extent of precipitation) in different locations of the country. Based on the learned knowledge in each location, we predict the frequency of future floods by searching for similar climate patterns in the future data. With no changes in the hydro-morphological regime of the region, we could expect a similar or worse climate condition could lead to similar or worse floods.

## Data and Statistics

**Figure 1:**
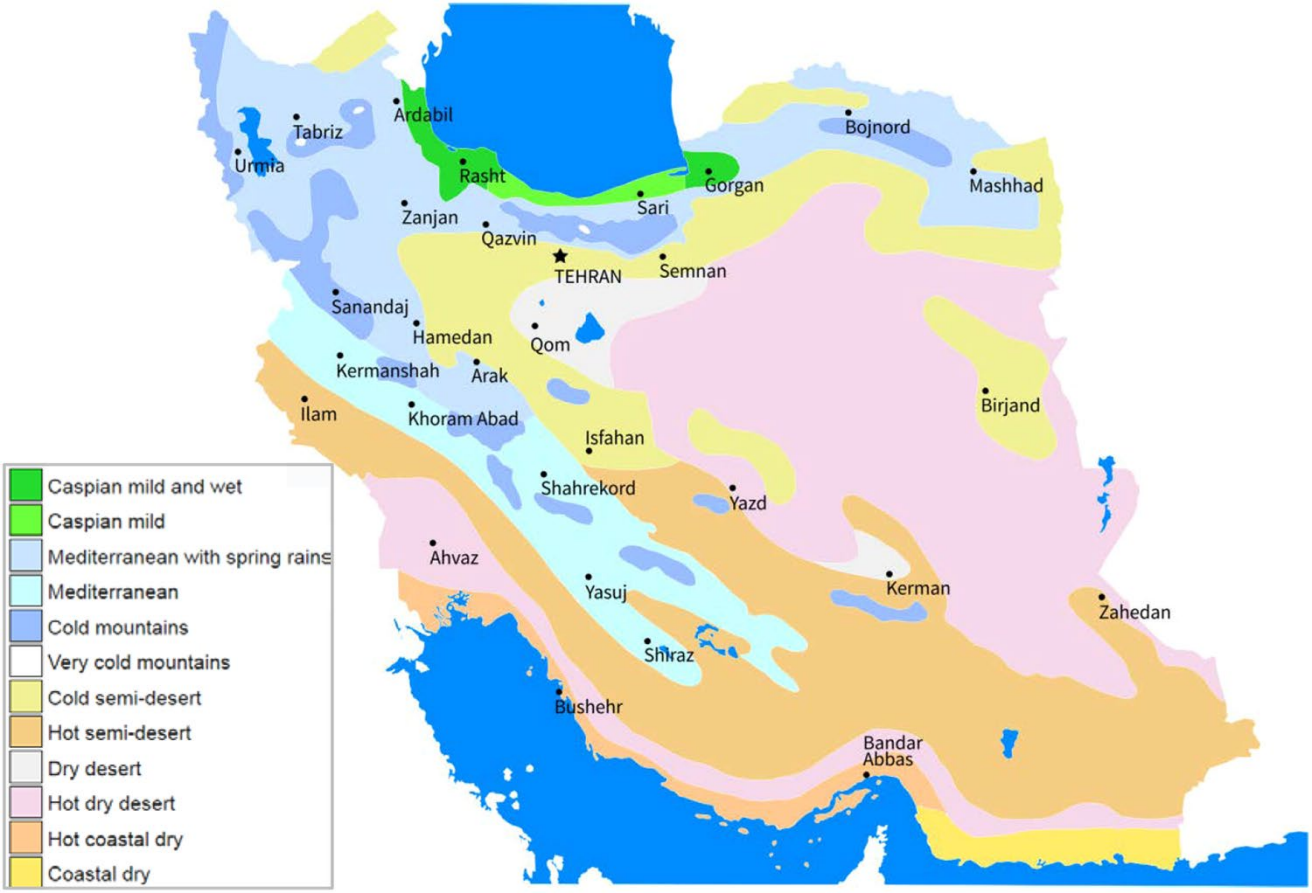
Climate map of Iran showing the location of province capitals^34^. This figure is shared under a CCBY 3.0 Share-Alike 3.0 Unported license and no changes were made to the original figure. https://creativecommons.org/licenses/by-sa/3.0/deed.en).

The elevation of Iran ranges from less than −28 m at the Caspian sea to 5,610 m in the Damavand peak of Alborz Mountain chain. Alborz Mountain in the north and Zagros Mountain in the west play a significant role in dissecting the country into various climatic zones. These mountainous regions block moisture to reach the central part of the country, which receives little rain and hosts one of the hottest deserts in the world, the Lut Desert. Approximately 88% of Iran is located in arid and semi-arid regions (Fig. 1)^34^. The mean annual precipitation during the study period (1980–2004) is 253 mm for the entire country, ranging from 144 mm to 342 mm per year. Iran receives less than a third of the world’s average precipitation^15^.

Despite the generally low precipitation, there have been large floods recorded in Iran. We used 6 floods, which occurred during 2015–2018, for the analysis of their future probable occurrences in this study (Table 1)^35,36^. Several physical factors are involved in the generation of floods such as slope, geology, landuse, soil, climate, and topographic wetness index^37^. It is thus often very difficult to quantify the contribution from individual variables^38^. Many model simulations, therefore, must be carried out with different variable settings^39^. In such cases, an ensemble of model runs needs to be executed for long time periods to reduce model uncertainty^40^.

**Table 1:** Historical flood conditions used in this study for future prediction^31,32^ (see Fig. 1 for locations in the country).

City/County	Province	Watershed	Flood Condition	Date	Area in km^2^ (No. of rain grids in the flooded basin)
(B) Ajabshir	East Azerbaijan	Urmia Lake	84 mm in 24 hrs	15–16 Apr 2017	19,045 (19)
(C) Azad-Shahr	Gorgan	Gorgan Rud	53 mm in 24 hrs	2 Sep 2016	10,416 (11)
(D) Iran-Shahr	Sistan & Balouchestan	Jazmoorian	86 mm in 4 days	28–31 Oct 2015	16,118 (11)
(E) Firooz-Abad	Fars	Mand	300 mm in 6 days	13–18 Feb 2017	39,879 (36)
(F) Abarkouh	Yazd	Abarghoo	20 mm in 24 hrs	4 May 2018	5,335 (5)
(G) Bushehr	Bushehr	Helleh	230 mm in 3 days	15–17 Feb 2017	11,133 (12)

To avoid such tedious and uncertain calculations, in this study we suggest a more straightforward approach for parameterizing a flood condition by using statistics of previous floods. In this approach, we quantify past floods considering the volume of water generated by precipitation at different locations within the flooded basin every day before flooding and project this data into the future by a search routine that identifies similar or worse conditions. We define the basin as the area that contributes to flooding water at its outlet (or flood point). In this calculation, we are not assuming that floods would not occur in any other way, but merely calculate a likely flood if similar conditions of a past flood or worse happen in the future (see Methods for more detail).

We cannot evaluate the future climate predictions by Atmosphere-Ocean General Circulation Models (GCMs) for their correctness. For this reason, it is necessary to run a large number of models and Representative Concentration Pathways (RCPs) to quantify the uncertainties. Flood and extreme climate studies have widely used GCM data^26–28,41^. In this study, we used four sets of climate data that included: (*i*) 122 observed station data (1980–2004)^42^, (*ii*) 0.5° gridded historical (1980–2004) data^43^, (*iii*) historical parts of five widely used global 0.5° GCM models (1980–2004) (Table 2)^44,45^, and (*iv*) the future simulations of the five GCM data (2025–2049). We used the observed data in (*i*) to bias correct the 0.5° gridded data in (*ii*) to obtain a uniformly distributed grid across the country. We then used the latter to bias correct the GCM data for RCP4.5 and RCP8.5 scenarios (see Figs S1–S3 in Supplementary Material for bias correction results). The grid data is often successfully used as observed data in places where sufficient observation is lacking^46,47^ (see Methods for more detail).

**Table 2:** IMO^42^ observed station data used to downscale the historical CFSR data (1980–2004).

Data	Scenario	Institute
IMO	Historical	Iranian Meteorological Organization, 122 Observed station data
CFSR	Historical	Climate Forecast System Reanalysis
GFDL-ESM2M (GCM1)	RCP(4.5, 8.5)	NOAA/Geophysical Fluid Dynamics Laboratory
HadGEM2-ES (GCM2)	RCP(4.5, 8.5)	Met Office Hadley Centre
IPSL-CM5A-LR (GCM3)	RCP(4.5, 8.5)	L’Institute Pierre-Simon Laplace
MIROC (GCM4)	RCP(4.5, 8.5)	AORI, NIES and JAMSTEC
NoerESM1-M (GCM5)	RCP(4.5, 8.5)	Norwegian Climate Center

CFSR^43^ historical data at 0.5° grid used as observed to downscale GCMs. Five Atmosphere Ocean General Circulation Models (GCMs)^44,45^ at 0.5° grid used in this research for two Representative Climate Pathways (1980–2049). RCP4.5 representing an average case and RCP8.5 representing an extreme situation.

## Results and Discussion

### Future maximum and minimum temperatures

**Figure 2:**
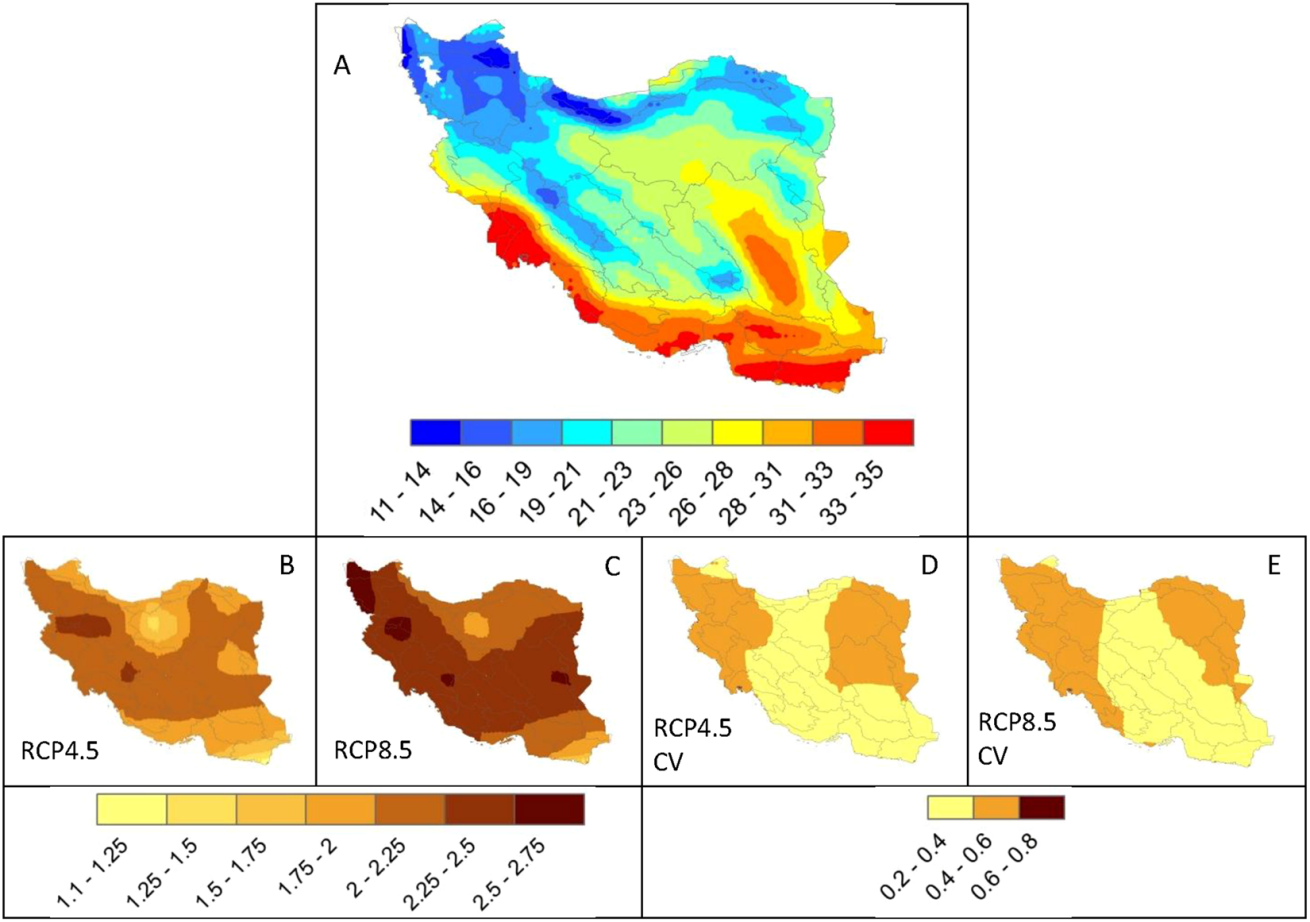
Maximum temperature distribution (°C). (**A**) illustrates the historical distribution. (**B,C**) are the differences between averages of historical and the 5 GCM ensembles. Increases of 1.1 to 2.75 °C are seen across the country. (**D,E**) illustrate the coefficient of variation of the 5 models. The larger the coefficient of variation, the larger the disagreement between the 5 models in each scenario. The models agree more in the central and southeastern parts of Iran across all climate zones.

The ensemble of 5 GCMs shows an increase of 1.1 to 2.75 °C in maximum temperature across Iran (Fig. 2). The two RCPs show similar patterns of change with differing magnitudes. RCP8.5 shows a sharp temperature rise of 2 to 2.75 °C in most parts of the country. The coefficients of variation show the degree of agreement between the five GCMs. As illustrated in Fig. 2, there are greater agreements in the models in the central to southeastern parts of the country, mostly in the Hot Dry Desert and Hot Semi-Desert climate zones. Results also indicate a moderate agreement in the west and the east in the primarily Mediterranean climate.

**Figure 3:**
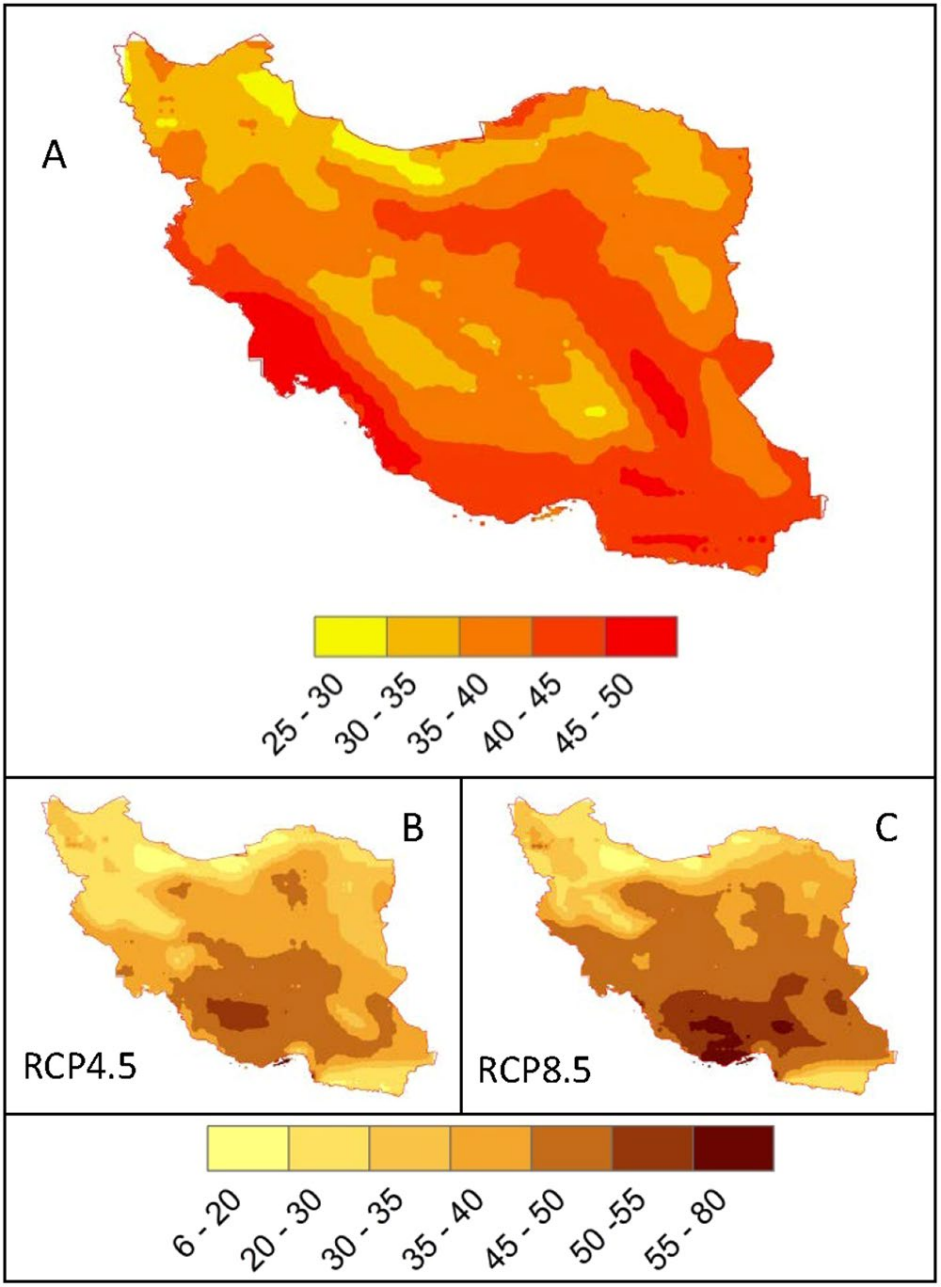
Extreme hot days for the historical and future periods. (**A**) is the 95^th^ percentile of historical maximum daily air temperature (Tmax) (°C) (1980–2004). The shown temperatures in (**A**) have occurred an average of 18 days per year. (**B,C**) show the number of days per year in the future (2025–2049) where extreme Tmax exceeds the historical value of 18 days. Most regions in Iran may experience longer extreme hot days of up to 2 months per year.

**Figure 4:**
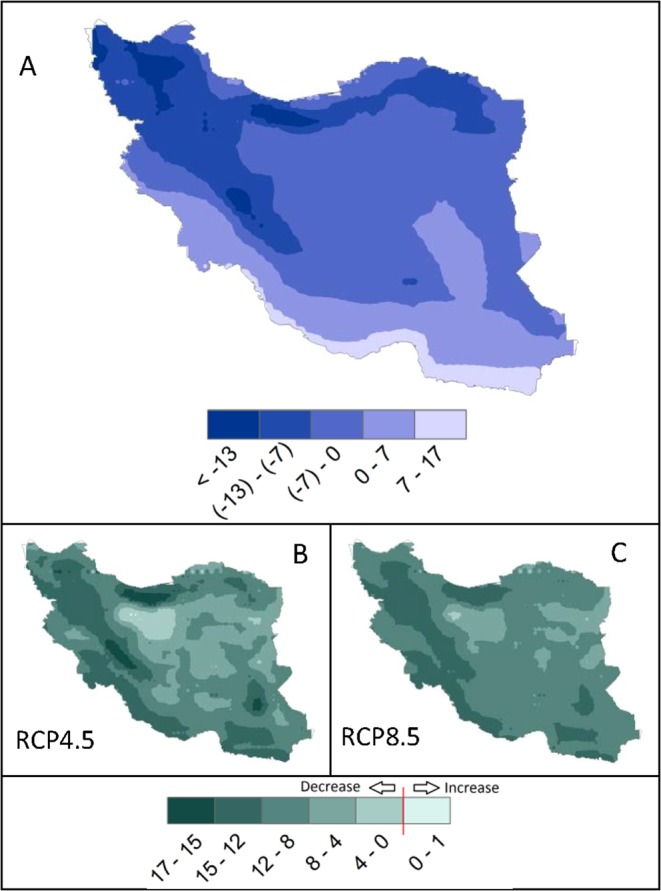
Extreme cold days for the historical and future periods. (**A**) is the 5^th^ percentile of historical minimum daily air temperature (Tmin) (°C) (1980–2004). The shown temperatures in (**A**) have occurred an average of 18 days per year. (**B,C**) show the number of days per year in the future (2025–2049) where extreme Tmin is less than the historical value of 18 days. Most regions in Iran may experience fewer extremely cold days per year.

Extreme temperatures are wreaking havoc in many countries around the world with an increasing number of deaths^48^. We calculated the 95^th^ percentile of maximum (Fig. 3 top) and 5^th^ percentile of minimum (Fig. 4 top) temperatures for the historical time at every climate grid across Iran. These temperature records occurred about 18 days per year. For the future, both climate scenarios predict a significantly higher 30–90 days per year of extremely hot temperatures in the Desert climates. Nonetheless, many regions across the country experience extremely hot temperatures of up to 30 days across different climate zones. The number of days of extreme cold temperature in the future, however, drops across the country and all areas are predicted to experience a fewer number of extreme cold temperatures (Fig. 4).

### Future precipitation

**Figure 5:**
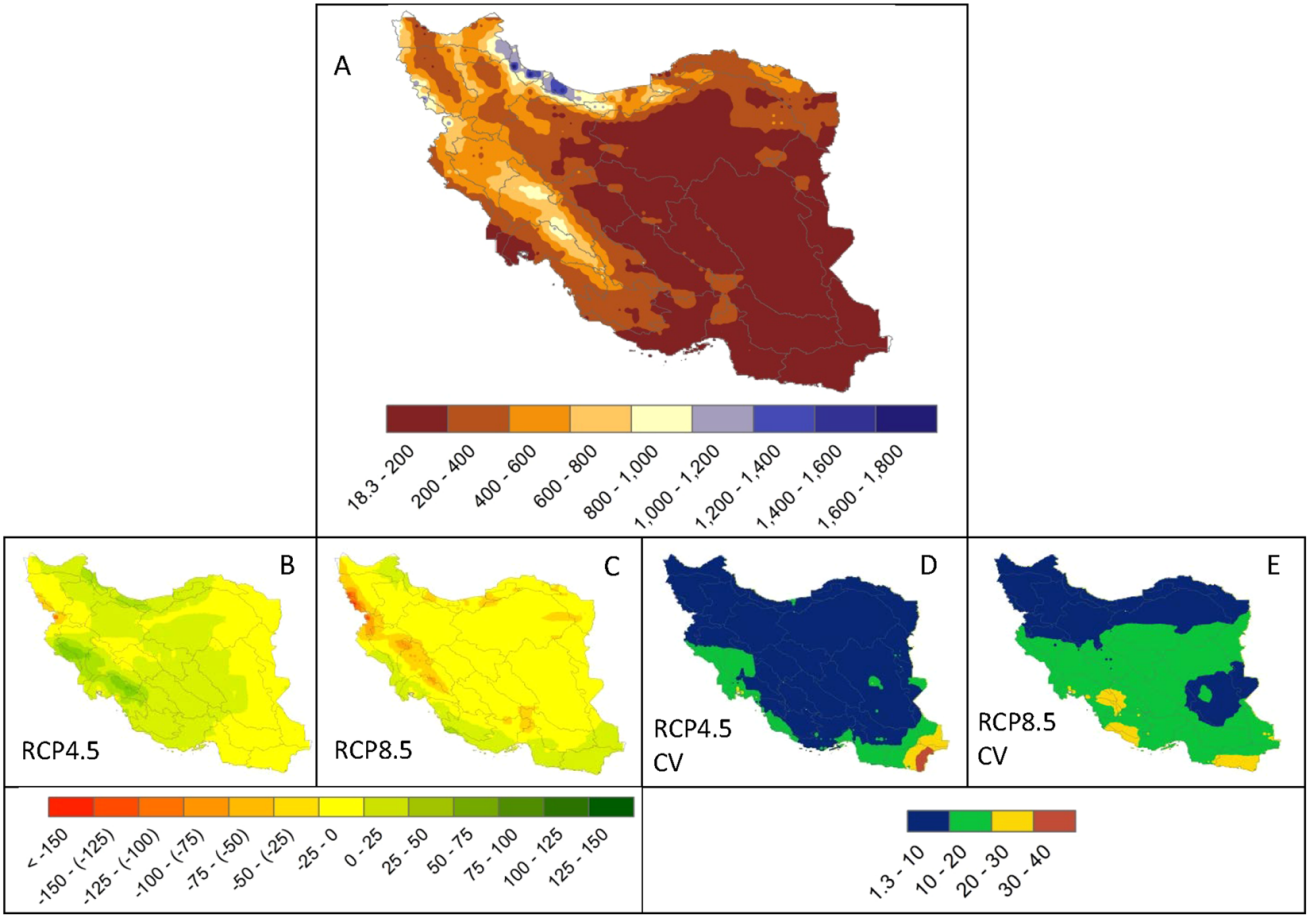
Rainfall distribution (mm). (**A**) illustrates the historical average annual rainfall distribution (1980–2004). (**B,C**) show the differences between the historical rainfall and the averages of the 5 GCMs. (**D,E**) illustrate the coefficient of variation in the 5-model ensemble. RCP4.5 shows high agreement between the models, while in RCP8.5 models agree more in the northern part of the country.

The ensemble of all models for both scenarios predicts no significant change in the entire country’s average annual precipitation during the future study period. However, spatially, there may be a considerable change of up to ±100 mm year^−1^ (Fig. 5). Unlike temperature, precipitation is projected quite differently by the two RCPs. RCP4.5 predicts a rise of up to 100 mm year^−1^ in the Mediterranean and Semi-Desert climates and the Caspian zones and a modest increase in the central Cold Semi-Deserts. In this scenario, similar to a previous study^29^, the wet regions of the country get wetter, while the dry areas get drier. In contrast, scenario RCP8.5 predicts a significant decrease in the precipitation of about 100 mm year^−1^ in the Hot Semi-Desert areas and a relatively stable Caspian zone climate.

### Compound analysis of dry and wet conditions

The compound analysis allows for consideration of several variables simultaneously. Here, we used the compound analysis to identify extreme dry and wet periods across Iran using precipitation and temperature. Compound extremes exert the most substantial impacts on the environment. To demonstrate, for an extremely dry period, we assumed a condition where (for ≥120 consecutive days: precipitation <2 mm day^−1^ and Tmax ≥30 °C), and for an extremely wet period, we assumed a condition where (for ≤3 consecutive days: the total amount of precipitation ≥110 mm). These conditions were chosen subjectively here and could differ for different regions. The compound analysis could include other variables such as soil moisture, humidity, evapotranspiration, or crop yield, subject to data availability.

**Figure 6:**
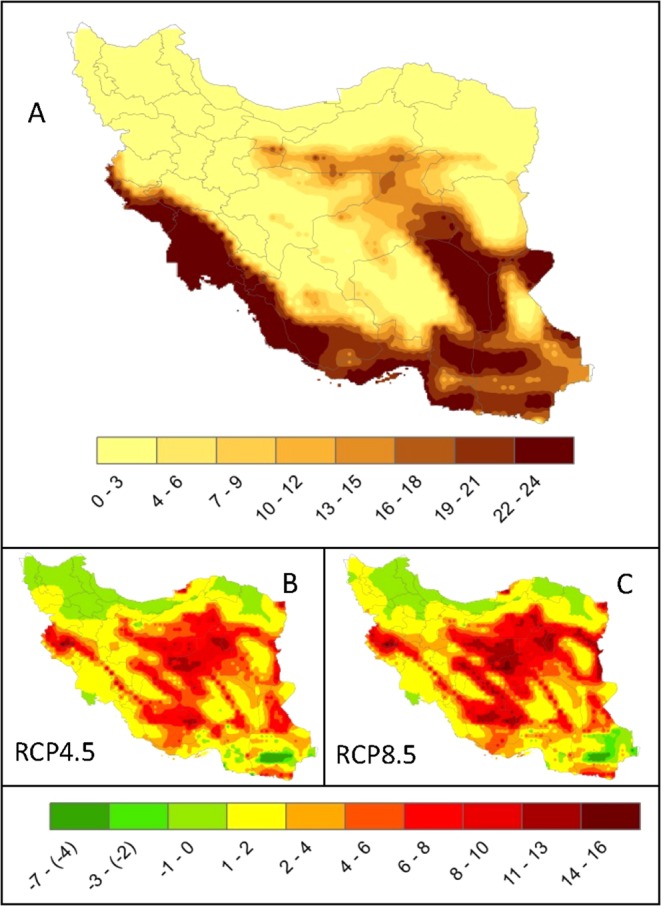
Compound analysis for dry periods where (for ≥120 consecutive days, rainfall <2 mm day^−1^ and Tmax ≥30 °C). (**A**) shows the frequency of such conditions in the past (1980–2004). (**B,C**) illustrate the difference between the historical and the future (2025–2049) frequencies of the events. Increasing extreme dry periods are predicted for Hot Dry Desert and Hot Semi-Desert areas, while Caspian Sea Mild and Wet zone may experience fewer extreme dry conditions in both scenarios.

Future analysis of extreme dry periods showed a significant 16-fold increase in most of the country south of the Alborz Mountain chain (Fig. 6). The increase in dry periods corresponds well with the increases in the extremely hot temperatures illustrated in Fig. 3. The Caspian and Wet Mediterranean zones in the north may, however, experience slightly fewer dry periods than before.

**Figure 7:**
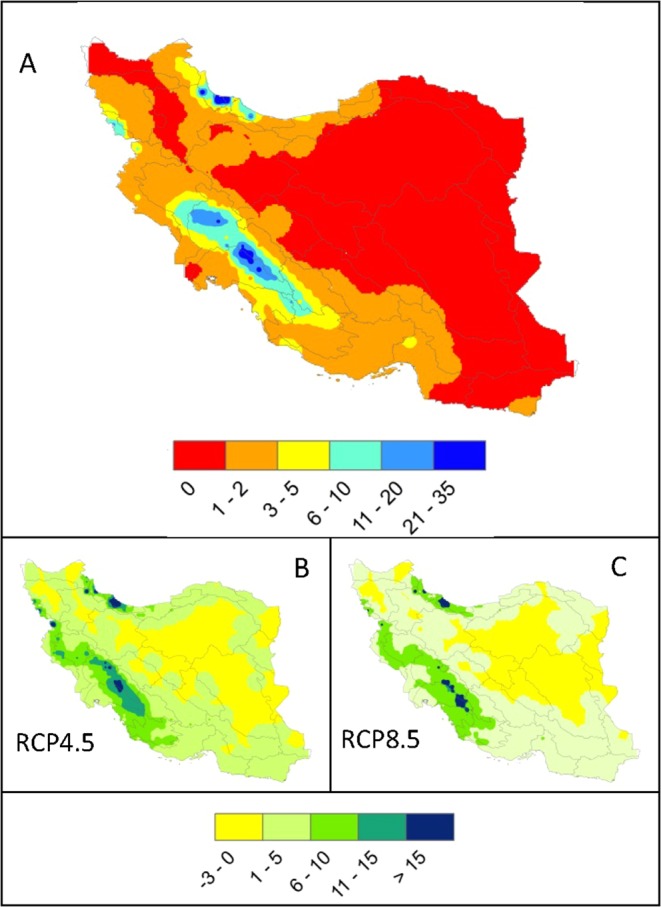
Compound analysis for wet periods where (for ≤3 consecutive days: the total amount of rainfall ≥110 mm). (**A**) shows the frequency of such conditions in the past (1980–2004). (**B,C**) illustrate the difference between the historical and the future (2025–2049) frequencies of the events. Increasing extreme wet periods are predicted for most regions except the Hot Dry Desert climate zone. Western provinces along Zagros Mountain in Hot Coastal Dry and Cold Mountain zones may be most vulnerable to wet-period conditions.

Except for the Hot Dry Desert, the rest of the country is also projected to experience significant increases in extremely wet periods (Fig. 7). The significant increases in both extremely hot and wet periods simultaneously indicate long dry periods intermittently interrupted with precipitations of high intensity and short duration, which is a recipe for a higher probability of drought and flood conditions.

### Future Floods

**Figure 8:**
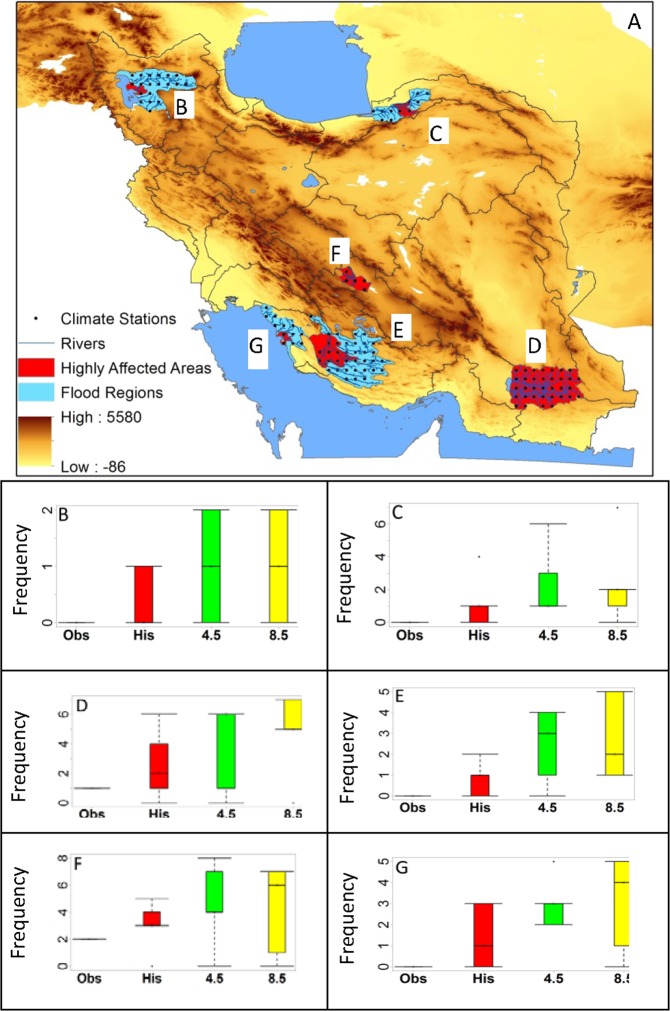
The top figure shows the 6 regions we considered for analysis in this paper for the period of (2025–2049) (see also Table 1). Blue areas in the map show the basin and red areas show the location of floods. Frequency of floods are shown in: (**B**) Ajabshir in East Azerbaijan province, (**C**) Azad-Shar county in Gorgan province, (**D**) Iran-Shar county in Sistan & Balouchestan province, (**E**) Firooz-Abad county in Fars province, (**F**) Abarkouh county in Yazd province, and (**G**) Bushehr county in Bushehr province. The largest increase is seen in the Lorestan province, which falls in the Mediterranean with Spring Rain climate zone.

We simulated the conditions of the most recent floods (Table 1) using three climate datasets: gridded observed (1980–2004), historical GCM (1980–2004), and future GCM (2025–2049). The close comparison of the number of floods in the gridded observed data versus the historical GCM attests to the adequacy of the flood model used in this study to flag up a probable flood. The future results show varying degrees of flooding increases in different parts of the country (Fig. 8). The most substantial increases in floods occur in the Desert regions in the southern parts of the country where despite decreasing precipitation, wet periods and the risk of floods could still increase in the future.

### What about natural climate variability?

Simulation of mid-latitude atmospheric blocking has always been a weak point of GCM models^49^. Although some improvements have been reported^49,50^, still considerable errors exist in GCMs as a result of blocking. Iran, because of its location in the mid-latitudes, is affected in different seasons by anticyclones and blocking patterns^51^ whose frequency can be expected to increase under conditions of global warming^52^. For instance, mountainous regions in western Iran (Zagros Mountains range) are influenced by the Mediterranean cyclones^53,54^, whereas southeastern fringes of Iran are affected by the Monsoon weather phenomena^55^. Also, El Niño-Southern Oscillation is reported to be responsible for intensification of March–April floods compared with normal conditions^56^. Blocking causes alteration of weather patterns leading to floods, droughts, unusual temperatures, and other weather extremes. The results reported in this study, therefore, should be taken as likely events under the ‘new normal’ future climate that could be modified by various occurrences of natural climate variability.

In summary, our study of future climate in Iran depicts a grim picture concerning more frequent extreme wet and dry periods, more extended periods of extremely hot temperatures, and higher frequency of floods across the country. Combination of these events, especially in the three Desert climate zones, may create an uninhabitable living condition as also suggested by other studies^48^. More resilient multipronged adaptive measures, therefore, must be taken to protect the people and the cities from these disasters.

## Methods

### Flood volume calculation

We first identified the flooded points, then used GIS to draw the contributing basin boundary. After determining the climate grids that fell in the corresponding basins, we calculated weighted-average precipitation multiplied by basin area to obtain the total volume of water in the basin going through the flooded region in a day using the following expressions:

1 $${V}_{j}={R}_{j}{A}_{j}$$

2 $${V}_{d}=\mathop{\sum }\limits_{j=1}^{n}{V}_{j}$$

3 $${V}_{T}=\mathop{\sum }\limits_{d=1}^{D}\mathop{\sum }\limits_{j=i}^{J}{V}_{d,j}$$

where *V*_*j*_ is volume of water generated by the *j*th climate grid in the basin in a day (m^3^), *R* is the precipitation (m day^−1^), *A* is the tributary area of a precipitation grid (m^2^), *V*_*d*_ is the volume of water generated in one day in the basin by precipitation from all grids (m^3^), and *V*_*T*_ is the total volume of water generated in *D* days of rain. If a historical flood occurred after *D* rainy days, then a future flooding condition is formulated as:

Future Flood = True, if:

$$({V}_{F,j}\ge {V}_{H,j},\forall \,j)\,{\rm{.AND}}{\rm{.}}\,({V}_{F,d}\ge {V}_{H,d},\forall \,d)\,{\rm{.AND}}{\rm{.}}\,({V}_{F,T}\ge {V}_{H,T})$$

where *F* and *H* stand for future and historical, respectively.

### Climate data

Climate data used in this study consisted of: (*i*) observed station data (122 stations, 1980–2004) (IMO)^42^, (*ii*) 0.5° gridded historical data from National Center for Environmental Prediction (NCEP) Climate Forecast System Reanalysis (CFSR)^43^ (1607 grid points inside the country, 1980–2004), (*iii*) historical part of five global 0.5° GCM models (1980–2004) that were included in the fifth phase of Coupled Model Intercomparison Project (CMIP5)^44^ and derived from ISI-MIP5 (Inter-Sectoral Impact Model Intercomparison Project)^45^ under four greenhouse gas concentration trajectories, i.e., Representative Concentration Pathways (RCP2.6, RCP4.5, RCP6.0 and RCP8.5), and (*iv*) the future part of the five GCM data for the four RCPs (2025–2049) (Table 2). Based on preliminary analyses and comparison tests, the CFSR data, after bias correction, provided a reasonable estimate of precipitation across the country.

First, we bias corrected the CFSR data based on the 122 observed metrological stations, then GCMs were bias-corrected based on corrected CFSR data. For bias correction and pattern recognition of a historical flood condition, we used the Climate Change Toolkit (CCT) program^57^.

A key aspect of the climate change impact study is the spatial and temporal downscaling of the GCM results. In this study, the GCM data were downscaled using the nearest observation station. For precipitation, we used a linear correction method. GCM daily precipitation amounts, *p*, were transformed into p* using a scaling factor α such that p* = α*p*, where $\alpha =\bar{O}/\bar{P},\bar{O}$, is the average monthly observed precipitation, and $\bar{P}$ is the average monthly GCM precipitation. Here, the monthly scaling factor was applied to each uncorrected daily observation of that month, generating the corrected future daily time series.

For temperature, we tested linear and nonlinear models as used in the literature^29,58,59^ and chose a fourth-degree regression model based on the calibration and validation results of stations in different regions. In general, the results of a first-degree linear and a fourth-degree nonlinear model were similar except for small and large temperature values^29^, where the nonlinear model performed systematically better, especially for the validation data set. Hence we opted for the nonlinear model expressed as:

$${\rm{Observed}}\,{\rm{Data}}=a+b\,{\rm{GCM}}+c\,{{\rm{GCM}}}^{2}+d\,{{\rm{GCM}}}^{3}+e\,{{\rm{GCM}}}^{4}$$

where *a* is regression constant and *b* to *e* are regression coefficients. We then used this transformation to correct the future GCMs. (see Figures S1–S3 in Supplementary Material for an example of bias correction result).

## Supplementary information

supplementary information

## Data Availability

All data used in this paper for Iran were extracted from global data, which are available at https://www.2w2e.com and can also be requested from the corresponding author.

## References

[1] Trenberth KE, Fasullo JT, Shepherd TG (2015). Attribution of climate extreme events. Nature Climate Change.

[2] Trenberth KE, Dai A, Rasmussen RM, Parsons DB (2003). The Changing Character of Precipitation. Bulletin of the American Meteorological Society.

[3] Lenderink G, van Meijgaard E (2008). Increase in hourly precipitation extremes beyond expectations from temperature changes. Nature Geoscience.

[4] Schuur EAG (2008). Vulnerability of Permafrost Carbon to Climate Change: Implications for the Global Carbon Cycle. BioScience.

[5] Vuille M (2008). Climate change and tropical Andean glaciers: Past, present and future. Earth-Science Reviews.

[6] Sharp M (2011). Extreme melt on Canada’s Arctic ice caps in the 21st century. Geophysical Research Letters.

[7] Douville H, Ribes A, Decharme B, Alkama R, Sheffield J (2012). Anthropogenic influence on multidecadal changes in reconstructed global evapotranspiration. Nature Climate Change.

[8] Abtew, W. & Melesse, A. *Evaporation and Evapotranspiration: Measurements and Estimations*, 197–202 (Springer Netherlands, 2013).

[9] Haerter JO, Berg P (2009). Unexpected rise in extreme precipitation caused by a shift in rain type?. Nature Geoscience.

[10] Singleton A, Ralf T (2013). Super-Clausius–Clapeyron scaling of precipitation in a model squall line. Quarterly Journal of the Royal Meteorological Society.

[11] Berg P, Moseley C, Haerter JO (2013). Strong increase in convective precipitation in response to higher temperatures. Nature Geoscience.

[12] Walther G-R (2002). Ecological responses to recent climate change. Nature.

[13] Zhang X (2011). Indices for monitoring changes in extremes based on daily temperature and precipitation data. Wiley Interdisciplinary Reviews: Climate Change.

[14] Alizadeh-Choobari O, Najafi MS (2018). Extreme weather events in Iran under a changing climate. Climate Dynamics.

[15] Madani K (2014). Water management in Iran: what is causing the looming crisis?. Journal of Environmental Studies and Sciences.

[16] Abbaspour M, Javid AH, Mirbagheri SA, Ahmadi Givi F, Moghimi P (2012). Investigation of lake drying attributed to climate change. International Journal of Environmental Science and Technology.

[17] Kaffashi S, Shamsudin MN, Radam A, Rahim KA, Yacob MR (2013). We are willing to pay to support wetland conservation: local users’ perspective. International Journal of Sustainable Development & World Ecology.

[18] Etemadi H, Samadi S, Sharifikia M (2014). Uncertainty analysis of statistical downscaling models using general circulation model over an international wetland. Climate Dynamics.

[19] Molle F, Hoogesteger J, Mamanpoush A (2008). Macro- and micro-level impacts of droughts: the case of the Zayandeh Rud river basin, Iran. Irrigation and Drainage.

[20] Gohari A (2013). Water transfer as a solution to water shortage: A fix that can Backfire. Journal of Hydrology.

[21] Döll P, Schmied HM, Schuh C, Portmann FT, Eicker A (2014). Global-scale assessment of groundwater depletion and related groundwater abstractions: Combining hydrological modeling with information from well observations and GRACE satellites. Water Resources Research.

[22] Richard D. Floodlist. http://floodlist.com/asia/iran-flash-floods-august-2017 (2018).

[23] Sharifi F, Samadi SZ, Wilson CAME (2012). Causes and consequences of recent floods in the Golestan catchments and Caspian Sea regions of Iran. Natural Hazards.

[24] Modarres R, Sarhadi A, Burn DH (2016). Changes of extreme drought and flood events in Iran. Global and Planetary Change.

[25] Modarres R, Ghadami M, Naderi S, Naderi M (2018). Future extreme precipitation change projections in the north of Iran. Meteorological Applications.

[26] Hirabayashi Y (2013). Global flood risk under climate change. Nature Climate Change.

[27] Déqué M (2007). Frequency of precipitation and temperature extremes over France in an anthropogenic scenario: Model results and statistical correction according to observed values. Global and Planetary Change.

[28] Orlowsky B, Seneviratne SI (2013). Elusive drought: uncertainty in observed trends and short- and long-term CMIP5 projections. Hydrol. Earth Syst. Sci..

[29] Abbaspour KC, Faramarzi M, Ghasemi SS, Yang H (2009). Assessing the impact of climate change on water resources in Iran. Water Resources Resarch.

[30] Balling RC, Keikhosravi Kiany MS, Sen Roy S, Khoshhal J (2016). Trends in Extreme Precipitation Indices in Iran: 1951-2007. Advances in Meteorology.

[31] Najafi MR, Moazami S (2016). Trends in total precipitation and magnitude–frequency of extreme precipitation in Iran, 1969–2009. International Journal of Climatology.

[32] Soltani M (2016). Assessment of climate variations in temperature and precipitation extreme events over Iran. Theoretical and Applied Climatology.

[33] Raziei T, Daryabari J, Bordi I, Pereira LS (2014). Spatial patterns and temporal trends of precipitation in Iran. Theoretical and Applied Climatology.

[34] Wikimedia Commons contributors, “File:Iran-climate.png,” Wikimedia Commons, the free media repository, https://commons.wikimedia.org/w/index.php?title=File:Iran-climate.png&oldid=224111575 (accessed September 20, 2018).

[35] Floodlist.com. Iran. http://floodlist.com/tag/iran (2018).

[36] Brakenridge, G. R. Global Active Archive of Large Flood Events, Dartmouth Flood Observatory, University of Colorado. https://floodobservatory.colorado.edu/Archives/index.html (2018).

[37] Tehrany MS, Pradhan B, Jebur MN (2015). Flood susceptibility analysis and its verification using a novel ensemble support vector machine and frequency ratio method. Stochastic Environmental Research and Risk Assessment.

[38] Stott PA (2010). Detection and attribution of climate change: a regional perspective. Wiley Interdisciplinary Reviews: Climate Change.

[39] Coumou D, Rahmstorf S (2012). A decade of weather extremes. Nature Climate Change.

[40] Allen MR, Stott PA (2003). Estimating signal amplitudes in optimal fingerprinting, part I: theory. Climate Dynamics.

[41] Willner SN, Otto C, Levermann A (2018). Global economic response to river floods. Nature Climate Change.

[42] IMO. Iranian Meteorological Organization. http://www.weather.ir (2009).

[43] Research Data Archive, NCEP Climate Forecast System Reanalysis (CFSR). https://rda.ucar.edu/pub/cfsr.html (2018).

[44] Taylor KE, Stouffer RJ, Meehl GA (2012). An Overview of CMIP5 and the Experiment Design. Bulletin of the American Meteorological Society.

[45] Hempel S, Frieler K, Warszawski L, Schewe J, Piontek F (2013). A trend-preserving bias correction &ndash; the ISI-MIP approach. Earth Syst. Dynam..

[46] Schuol J, Abbaspour KC, Yang H, Srinivasan R, Zehnder AJB (2008). Modeling blue and green water availability in Africa. Water Resources Research.

[47] Monteiro JAF, Strauch M, Srinivasan R, Abbaspour KC, Gücker B (2016). Accuracy of grid precipitation data for Brazil: application in river discharge modelling of the Tocantins catchment. Hydrological Processes.

[48] Pal JS, Eltahir EAB (2016). Future temperature in southwest Asia projected to exceed a threshold for human adaptability. Nature Climate Change.

[49] Davini P, D’Andrea F (2016). Northern hemisphere atmospheric blocking representation in global climate models: Twenty years of improvements?. Journal of Climate.

[50] Guilyardi E (2012). New Strategies for evaluating ENSO Processes in climate models. Bulletin of the American Meteorological Society.

[51] Zarrin A, Ghaemi H, Azadi M, Farajzadeh M (2010). The spatial pattern of summertime subtropical anticyclones over Asia and Africa: A climatological review. Int. J. Climatology.

[52] Mokhov II, Semenov VA (2016). Weather and climate anomalies in Russian regions related to globalclimate change. Russian Meteorology and Hydrology.

[53] Alijani B (2002). Variation of 500 hPa flow patterns over Iran and surrounding areas and their relationship with the climate of Iran. Theoretical Applied Climatolgy.

[54] Azizi G, Arsalani M, Bräuning A, Moghimi E (2013). Precipitation variations in the central ZagrosMountains (Iran) since A.D. 1840 based on oak tree rings. Palaeogeography, Palaeoclimatology, Palaeoecology.

[55] Yadav RK (2016). On the relationship between Iran surface temperature and northwest India summer monsoon rainfall. International Journal of Climatology.

[56] Saghafian B, Haghnegahdar A, Dehghani M (2017). Effect of ENSO on annual maximum floods and volume over threshold in the southwestern region of Iran. Hydrological Sciences Journal.

[57] Vaghefi SA, Abbaspour N, Kamali B, Abbaspour KC (2017). A toolkit for climate change analysis and pattern recognition for extreme weather conditions – Case study: California-Baja California Peninsula. Environmental Modelling & Software.

[58] Wilby RL, Wigley TML (1997). Downscaling general circulation model output: a review of methods and limitations. Progress in Physical Geography.

[59] Vaghefi AS, Mousavi SJ, Abbaspour KC, Srinivasan R, Arnold JR (2015). Integration of Hydrologic and Water Allocation Models in Basin-Scale Water Resources Management Considering Crop Pattern and Climate Change: Karkheh River Basin in Iran. Regional Environmental Change.

