# Author Correction: The future of extreme climate in Iran

**DOI:** 10.1038/s41598-019-53784-0

**Published:** 2019-11-19

**Authors:** Saeid Ashraf Vaghefi, Malihe Keykhai, Farshid Jahanbakhshi, Jaleh Sheikholeslami, Azadeh Ahmadi, Hong Yang, Karim C. Abbaspour

**Affiliations:** 1 Eawag, Swiss Federal Institute of Aquatic Science and Technology, Duebendorf, Switzerland; 2 Department of Civil Engineering, Isfahan University of Technology, Isfahan, Iran; 3 Department of Watershed Management Engineering, Yazd University, Yazd, Iran; 4 Department of Environmental Science, University of Basel, Basel, Switzerland

Correction to: *Scientific Reports* 10.1038/s41598-018-38071-8, published online 06 February 2019

The original version of this Article contained a typographical error in the spelling of the author Azadeh Ahmadi, which was incorrectly given as Azade Ahmadi. This has now been corrected in the PDF and HTML versions of the Article, and in the accompanying Supplementary Information file.

