# Supplementary material for: The future of extreme climate in Iran
Source: Sci Rep. 2019 Feb 6;9:1464. doi: 10.1038/s41598-018-38071-8 (PMC6365571; doi:10.1038/s41598-018-38071-8)
Supplement: Supplementary file 1 — supplementary information [file 41598_2018_38071_MOESM1_ESM.docx]

**The future of extreme climate in Iran**

Saeid Ashraf Vaghefi, Malihe Keykhai, Farshid Jahanbakhshi, Jaleh Sheikholeslami, Azadeh Ahmadi, Hong Yang, Karim C. Abbaspour


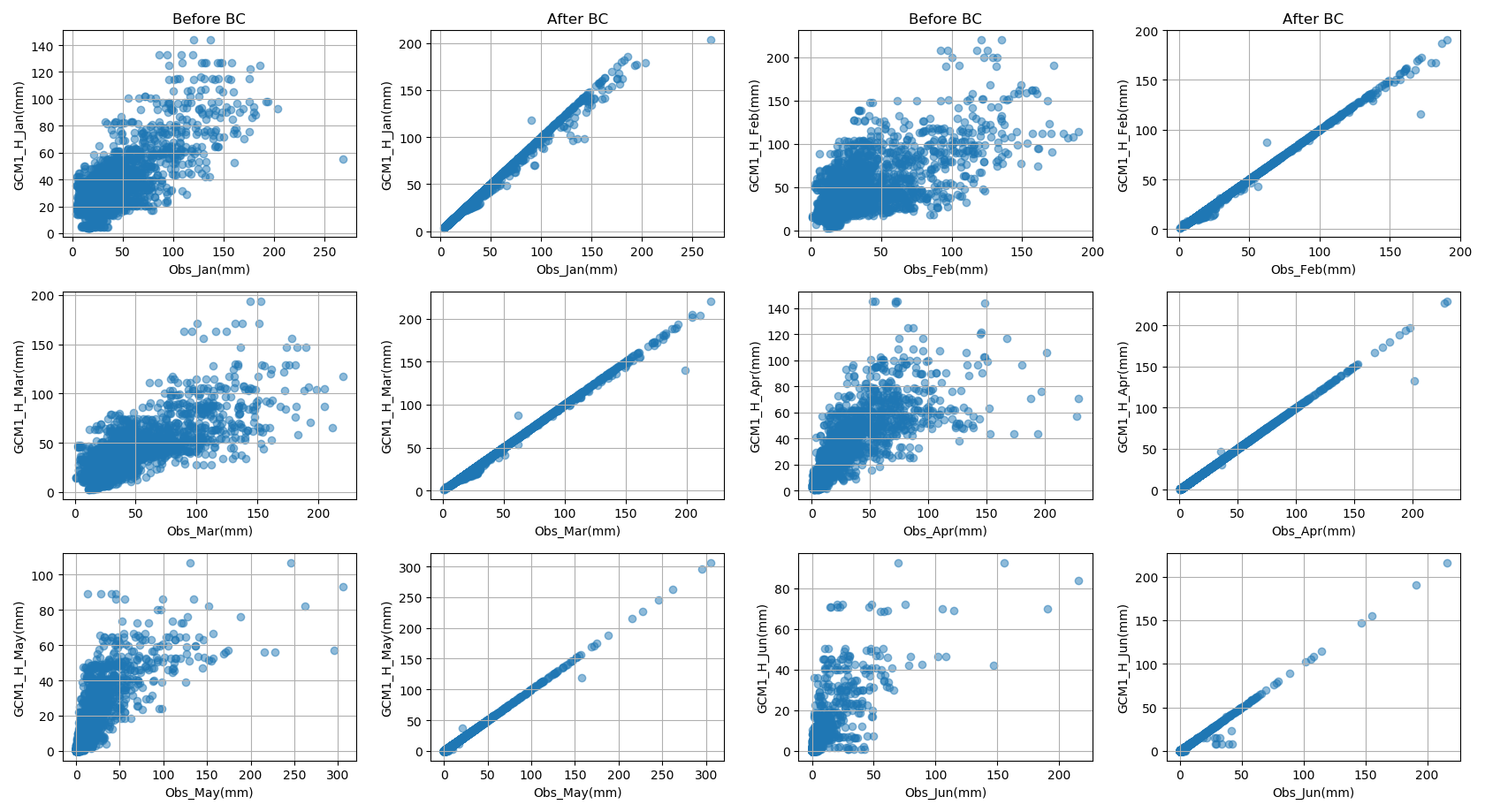

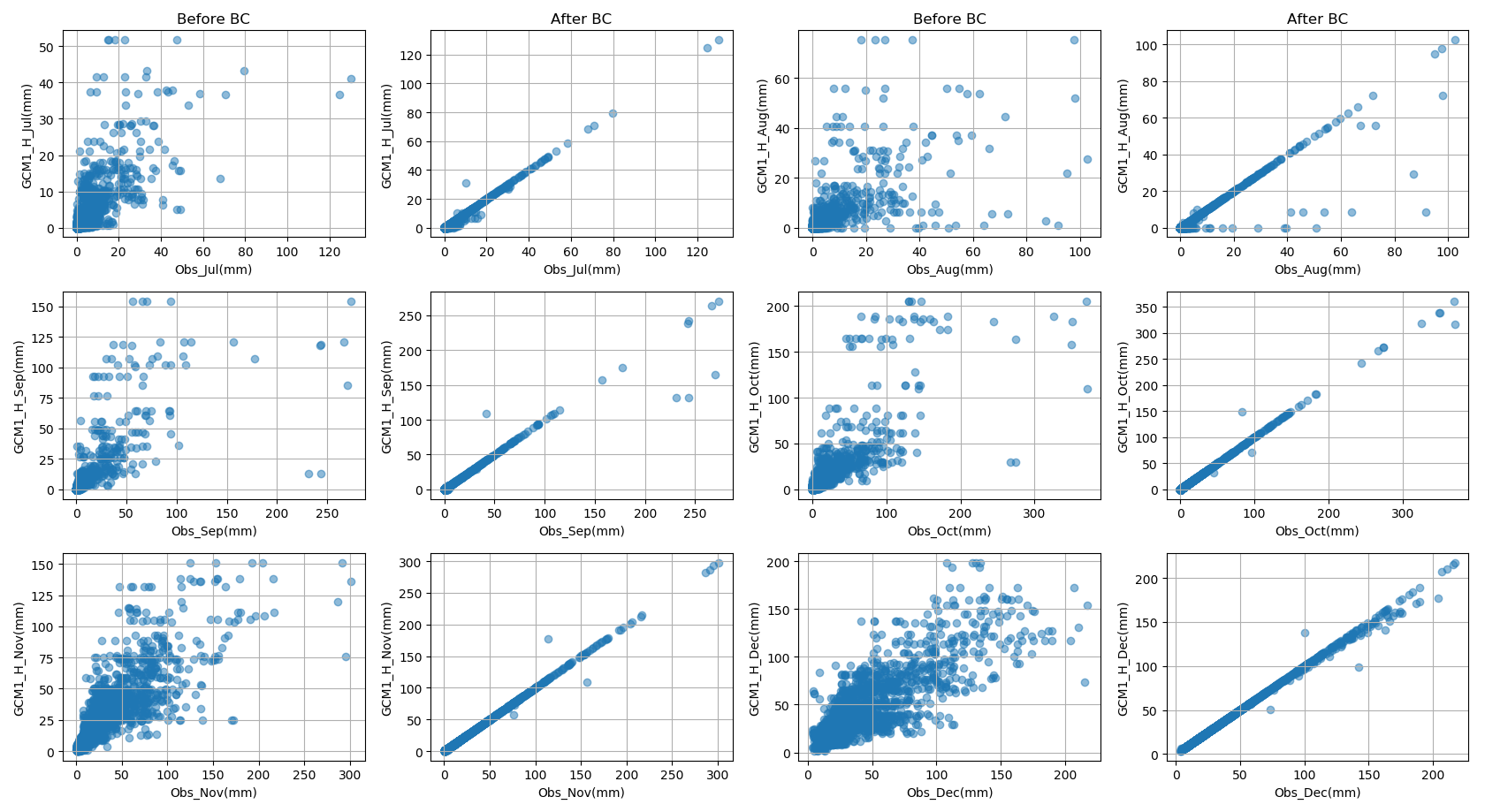


Figure S1. Comparison of monthly precipitation between observed data and GCM1 historical scenario before and after applying bias correction (ratio method). H:historic, BC:bias corrected


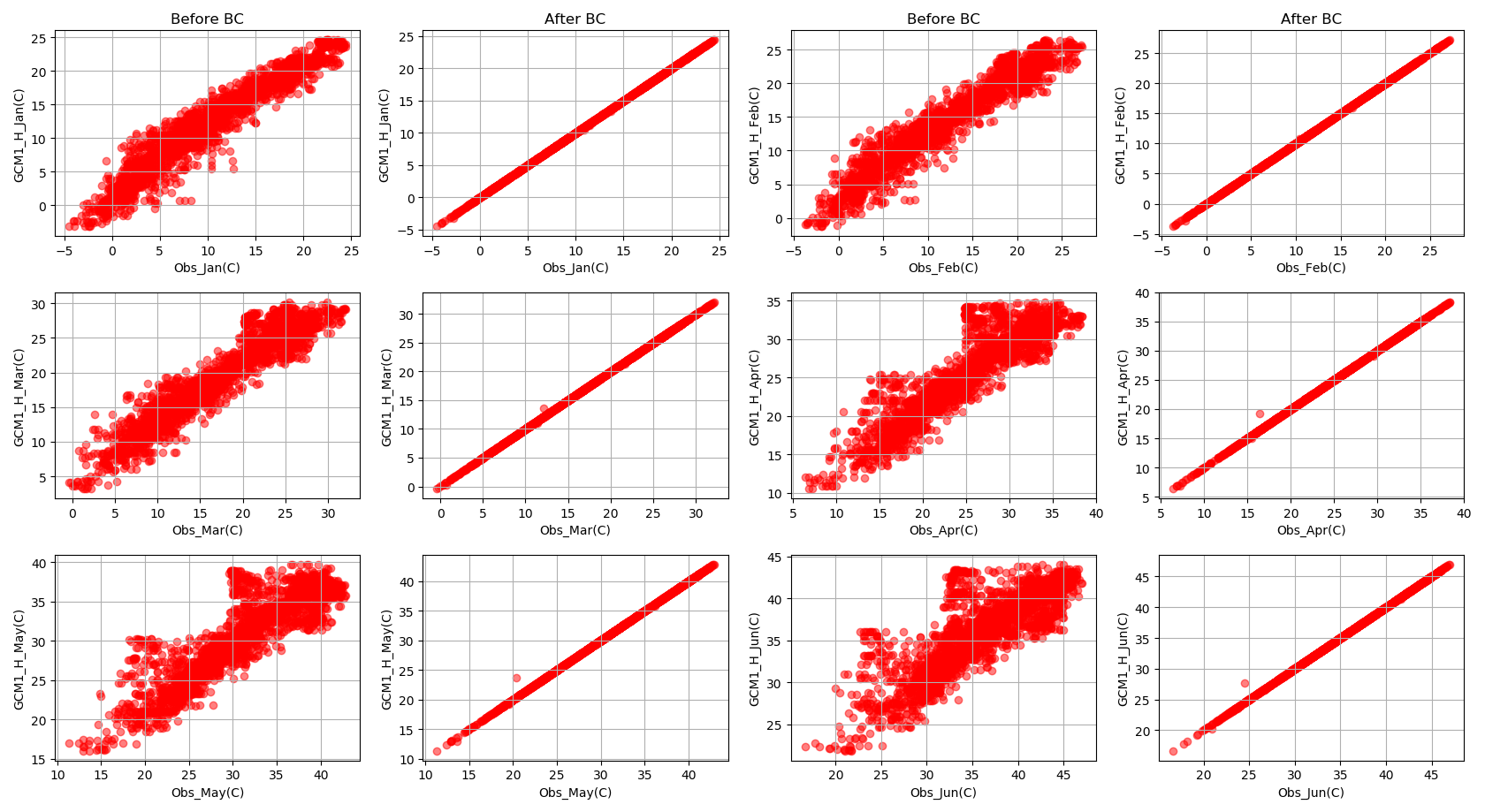

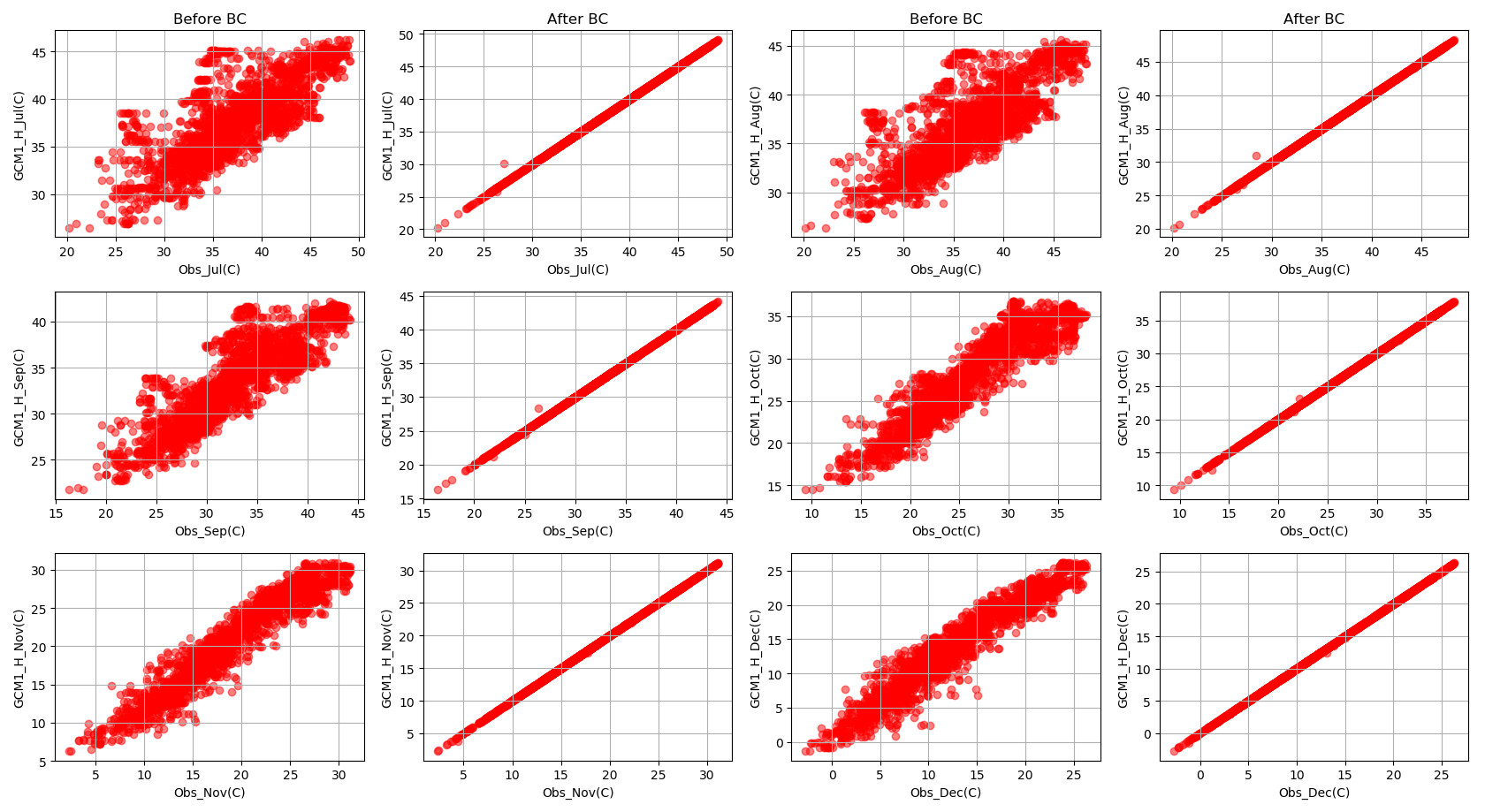


Figure S 2. Comparison of monthly maximum temperature between observed data and GCM1 historical scenario before and after applying bias correction (additive method). H:historic, BC:bias corrected


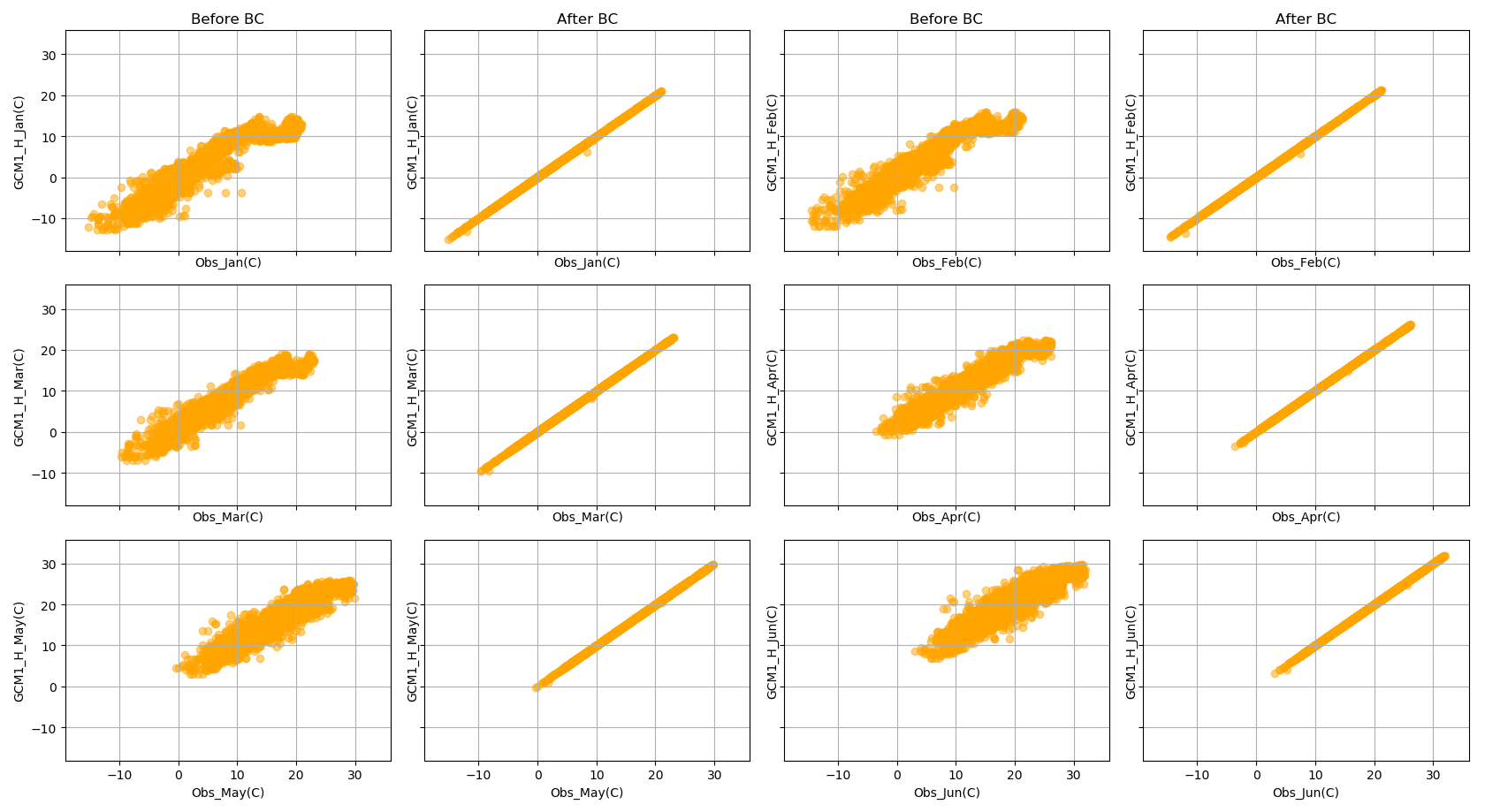

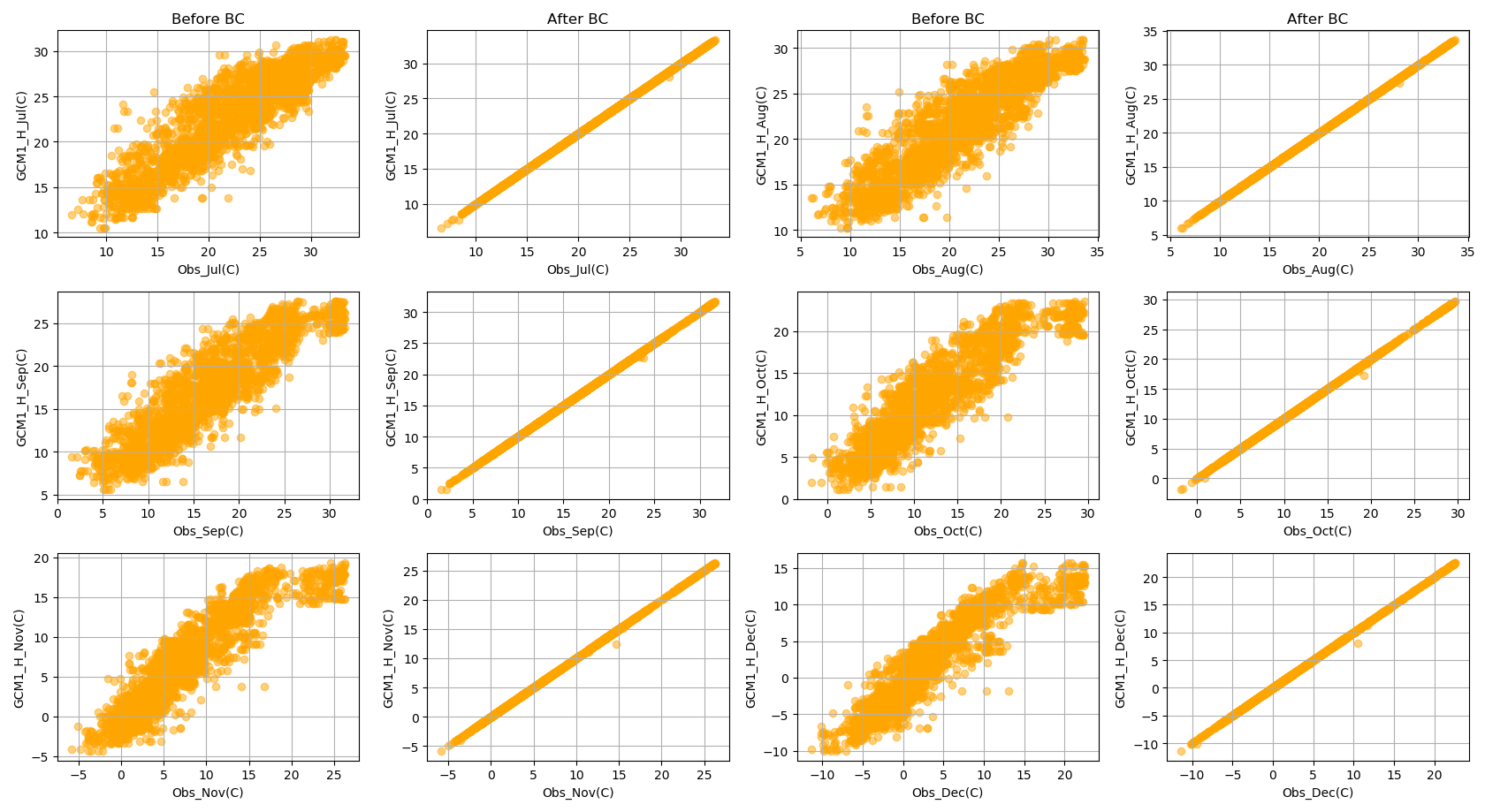


Figure S3. Comparison of monthly minimum temperature between observed data and GCM1 historical scenario before and after applying bias correction (additive method). H:historic, BC:bias corrected
